# Advancing Wearable-Based Upper-Limb Stroke Recovery Assessment to the Clinic: A Comparison of Movement Segmentation Strategies

**DOI:** 10.1109/TNSRE.2025.3635677

**Published:** 2026

**Authors:** Yunda Liu, Benito Lorenzo Pugliese, Gloria Vergara-Diaz, Anne O’Brien, Randie Black-Schaffer, Paolo Bonato, Sunghoon Ivan Lee

**Affiliations:** Manning College of Information and Computer Sciences, University of Massachusetts Amherst, Amherst, MA 01002 USA; Department of Physical Medicine and Rehabilitation, Harvard Medical School, Spaulding Rehabilitation Hospital, Boston, MA 02115 USA; Department of Physical Medicine and Rehabilitation, Harvard Medical School, Spaulding Rehabilitation Hospital, Boston, MA 02115 USA; Department of Physical Medicine and Rehabilitation, Harvard Medical School, Spaulding Rehabilitation Hospital, Boston, MA 02115 USA; Department of Physical Medicine and Rehabilitation, Harvard Medical School, Spaulding Rehabilitation Hospital, Boston, MA 02115 USA; Department of Physical Medicine and Rehabilitation, Harvard Medical School, Spaulding Rehabilitation Hospital, Boston, MA 02115 USA; Manning College of Information and Computer Sciences, University of Massachusetts Amherst, Amherst, MA 01002 USA

**Keywords:** Stroke, upper-limb rehabilitation, motor recovery, wearable sensors, movement segmentation

## Abstract

Continuous, objective, and precise upper-limb motor assessments are essential for realizing the vision of precision rehabilitation for stroke survivors. Wearable inertial sensors have emerged as a promising solution, enabling the analysis of motor performance in real-world settings. Recent studies have introduced two movement segmentation methods—anatomical segmentation and linear segmentation—for processing wearable inertial data to monitor post-stroke upper-limb motor recovery, each grounded in distinct theories of motor control and behavior. These methods differ in their practical implications for clinical use: linear segmentation requires only a single wearable device on the stroke-affected wrist, while anatomical segmentation necessitates an additional sensor on the sternum. This study seeks to systematically compare the clinimetric performance of these two approaches, taking into account their differences in practicality, to provide insights into their effective integration into clinical practice. 17 stroke survivors were equipped with inertial sensors on the trunk and the stroke-affected wrist while performing activities of daily living in a simulated apartment setting. Acceleration time-series from wrist movements were decomposed into movement segments using each movement segmentation approach. Reliable features were extracted from the movement segments, and supervised regression models were trained to establish concurrent validity against existing clinical measures. Anatomical segmentation demonstrated strong concurrent validity against existing clinical measures but may face challenges for continuous use due to the need for multiple sensors. Linear segmentation, on the other hand, provided slightly reduced but acceptable performance in motor deficit assessment while offering the advantage of requiring only a single wrist-worn sensor.

## I. Introduction

Stroke is a prevalent brain injury in the United States, with an estimated 9.4 million people reported to have had a stroke and approximately 795,000 new and recurring cases each year [1]. Motor impairment in the upper limbs is a common symptom post-stroke [2]. The impairment mainly manifests as the loss or reduced function in muscle control and movements [2], [3]. Stroke is a major cause of disability [4], significantly limiting patients’ ability to perform activities of daily living (ADLs) [5].

Frequent assessment of stroke survivors is essential as it provides a comprehensive understanding of the upper-limb motor recovery progress and informs strategies to enhance rehabilitation [2], [6]. Tools like the Fugl-Meyer Assessment for Upper-Extremity (FMA-UE) [7] and Wolf Motor Function Test (WMFT) [8] have been developed to assess *motor deficits* in upper-limb motor behaviors caused by the damages in the brain’s motor neuron networks. However, these tests are time-consuming and require trained specialists, making them impractical for routine use in the clinic [9]. These limitations have been recognized as a significant barrier, preventing rehabilitation specialists from regularly evaluating the need to adjust intervention to maximize motor gains [9].

To mitigate these challenges, researchers have explored the use of wearable sensors for objective, low-burden upper-limb motor assessment [10], [11]. By having stroke survivors wear sensors on their wrists and engaging in ADLs, researchers can extract objective information pertinent to the patient’s responses to interventions and their motor recovery process. Unfortunately, research on wearable-based monitoring to date has predominantly focused on evaluating *limb activity levels*, which aims to quantify the intensity of movements in the stroke-affected limb, or its comparison to the less-affected limb, by accumulating wrist acceleration magnitudes [12]. While these measures provide valuable insights into the extent of upper limb engagement during ADLs, they have shown limited concurrent validity and responsiveness to established measures of motor deficits, such as the FMA-UE, primarily attributed to the learned non-use phenomenon [13], [14].

To address this gap, recent studies have introduced a unique inertial data processing technique known as *movement segmentation*, designed to extract information more closely related to underlying motor deficits in the upper limb. This technique has been applied not only to stroke [15], [16] but also to other neurological conditions, such as cerebellar ataxia [17], amyotrophic lateral sclerosis (ALS) [18], and Parkinson’s disease [19]. The fundamental concept of this technique is to decompose continuous three-dimensional wrist inertial data into lower-level units of motor behaviors, called *movement segments*,^1^ using velocity zero-crossings [15]. Prior studies have shown that kinematic and temporal analysis of these movement segments allows for the extraction of information highly relevant to underlying motor deficits.

There are two major approaches to implementing the movement segmentation technique, each grounded in distinct theories of motor control and behavior: 1) linear segmentation and 2) anatomical segmentation. The linear segmentation method focuses on analyzing kinematic and temporal dynamics of movement segments during linear movements [22], [23]. Linear movements are characterized by straight and smooth wrist trajectories between two positions in 3D space [24], such as reaching-to-grasp, transporting, and retrieving-from-grasp movements [25]. Prior studies have analyzed the instantaneous velocity profiles of these linear movements and shown that healthy individuals exhibit consistent bell-shaped patterns [26], [27]. Under the premise that altered wrist kinematic patterns in individuals with brain damage or neurological conditions contain important information related to motor deficits, a line of research has focused on detecting the presence of linear movements, extracting movement segments, and analyzing their kinematic and temporal characteristics to assess underlying motor deficits [11], [17], [18], [21]. This approach offers the convenience of leveraging data from a single inertial sensor on the stroke-affected wrist, the most preferred location for long-term and continuous health monitoring [28], but the estimation of motor deficits relies on the accurate detection of linear movements among continuous and naturalistic upper-limb movements.

The anatomical segmentation method, on the other hand, is based on a distinct theory that complex 3D wrist movements, whether linear or non-linear, are planned and controlled independently along each of the body’s anatomical axes [20], [29]. This theory is supported by findings that movement segment velocity profiles, obtained by segmenting continuous wrist velocity using zero crossings within a Cartesian coordinate system aligned with anatomical planes (i.e., facing direction), also yield consistent bell-shaped patterns [20]. Prior research has shown that altered 3D velocity profiles of the wrist within the body coordinate system during naturalistic upper-limb movements contain information relevant to motor deficits [15], [17], [19], [30]. While this approach allows for the analysis of all types of upper-limb movements beyond linear motions, it requires two sensors—i.e., one on the wrist to capture wrist movements and another on the chest to capture the body axes—thereby reducing its overall usability.

Therefore, these two segmentation methods present a clear trade-off between the complexity of data analysis and usability, which respectively affects the clinimetric properties and practical applicability of the motor assessment. However, no systematic comparison has been conducted to compare their clinimetric properties, including reliability and concurrent validity.

In this paper, we investigate, for the first time to the best of our knowledge, this trade-off from a translational research perspective. Using data collected from 17 stroke survivors who performed ADLs naturally within a simulated home environment, we construct wearable-based upper-limb motor assessments utilizing two different movement segmentation approaches. We then evaluate and compare the reliability and concurrent validity of these assessments and discuss their respective benefits and limitations.

## II. Methods

### A. Study Participants

Data from 17 stroke survivors (59.9 ± 12.3 years old; mean ± standard deviation, 12 male) were collected and analyzed. To be included in the study, study participants 1) must be between 18 and 80 years old, 2) have a history of stroke at least 6 months prior to data collection, and 3) exhibit mild-to-moderate impairment in the upper limb affected by the stroke. The study was approved by the Mass General Brigham’s Institutional Review Board (IRB #2019P002612).

During the visit, trained clinicians evaluated participants’ motor deficits in the stroke-affected upper limb using the FMA-UE for motor impairment and the WMFT for functional ability. For the WMFT, both the Performance Time (WMFT-PT) and Functional Ability Score (WMFT-FAS) were obtained. Additionally, we collected patients’ perceived levels of functional performance using the Amount of Use (AoU) and Quality of Movement (QoM) scales of Motor Activity Log (MAL) [31]. Detailed clinical assessment scores, as well as information on limb dominance and laterality, are available in Supplementary Materials Table S1.

### B. Data Collection

Each subject wore Inertial Measurement Units (IMUs) (Shimmer3, Shimmer Research Inc., Ireland) on the stroke-affected wrist and the sternum. The IMUs sampled three-axis acceleration and three-axis angular velocity at a frequency of 50 *Hz*. Study participants were asked to perform a set of 11 pre-defined ADLs (see Table I) in a simulated environment, independently and in a naturalistic manner. These motor tasks were chosen to encompass a wide range of upper-limb movements, requiring varying degrees of gross arm and fine hand function, and were designed to closely replicate everyday activities. The selected tasks are consistent with those used in previous studies validating wearable devices for upper-limb performance analysis [32], [33]. During the experiment, each participant received a written list of ADLs along with guidance on locating necessary items to perform the tasks within the simulated environment. To preserve the naturalistic context, participants completed the tasks independently without step-by-step instructions from research staff. They were free to skip tasks that did not match their typical daily routines or that exceeded their current motor abilities, as well as to choose the order in which to complete the tasks. Each task could be repeated up to three times to capture within-subject variability in motor patterns, although the exact number of repetitions varied by task and participant. On average, 10.9 ± 0.8 (mean ± standard deviation) ADLs were performed by each participant. The data collection had an average duration of 49.7 ± 10.4 minutes.

To understand the context of the inertial data, four cameras were set up to record study participants’ motor performance: two egocentric cameras were strapped to the participant’s head and sternum, and two exocentric cameras were placed in the room. Video data were manually synchronized to the IMU data using annotation software (ELAN, The Language Archive, The Netherlands).

### C. Overview of Wearable-Based Motor Assessments

**Fig. 1.**
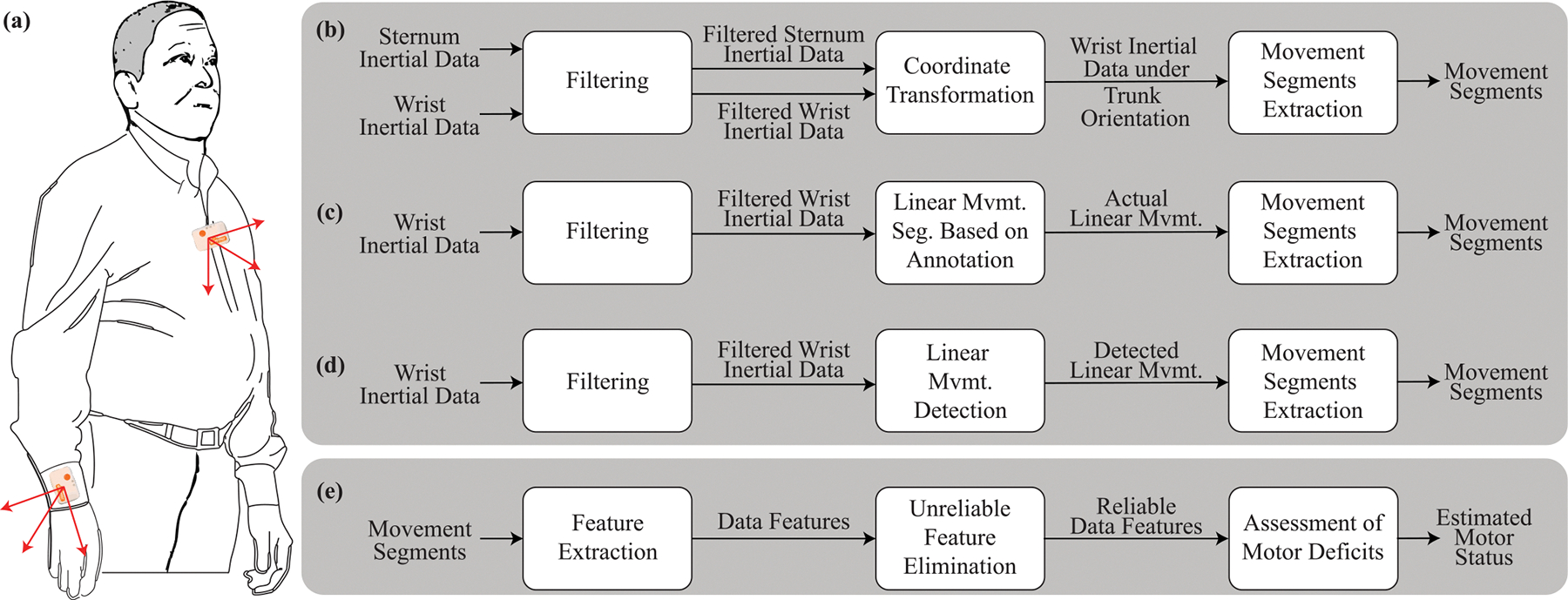
The plot shows data analytic pipeline used to establish wearable-based upper-limb motor assessments employing two different movement segmentation methods. (a) A subject wore two IMUs on the stroke-affected wrist and the sternum. (b) The pipeline for anatomical segmentation method. (c)-(d) The pipeline for linear segmentation method where the linear movements are created through manual annotation and detected by CNN, respectively. (e) The pipeline for motor deficit assessment using movement segments.

We developed wearable-based motor assessment tools using movement segments derived from both anatomical and linear segmentation methods, as summarized in Fig. 1. For the linear segmentation method, we identified linear movements using two different methods: 1) actual linear movements performed by study participants, manually annotated by research staff via video reviews using the definitions proposed by Schambra et al. [25], and 2) linear movements detected by a Convolutional Neural Network (CNN) trained on the manual annotation. The manually annotated linear movements were used to establish the theoretical upper bound of the linear segmentation method’s performance. In contrast, the automatically detected linear movements, which may contain detection errors, were evaluated to assess their impact on the integrity of the digital motor assessment, an essential consideration given that manual annotation is time-intensive for practical implementation. Fig. 1b–d illustrate the data analytic pipelines for these movement segmentation methods.

Movement segments derived from these three approaches—anatomical segmentation, linear segmentation with annotated linear movements, and linear segmentation with detected linear movements—were processed independently to produce a comprehensive measure quantifying motor deficits, as shown in Fig. 1e. This process involves feature extraction, unreliable feature elimination, and machine learning training. The resulting assessment of motor deficits were then compared in terms of reliability and concurrent validity.

### D. Inertial Data Pre-Processing

To attenuate high-frequency, non-human generated noise in the IMU data, the acceleration and angular velocity were low-pass filtered using a 6^*th*^ order Butterworth filter with a cut-off frequency of 10 *Hz* [34]. In our study, magnetometer data were excluded from sensor orientation estimation as they could be susceptible to electromagnetic noise in real-world environments [35]. The filtered acceleration and angular velocity from each sensor’s local coordinates (i.e., $L_{w}$ for wrist and $L_{s}$ for sternum) were fused to estimate the sensor’s orientation in the global coordinates (G) where the z-axis is aligned with gravity [36]. Suppose $q_{L_{w}}^{G}$ and $q_{L_{s}}^{G}$ represent the orientation of the wrist and sternum sensors in the global coordinates, respectively. With $q_{L_{w}}^{G}$, the wrist acceleration in the local coordinate $a_{w}^{L_{w}}$ was transformed into the acceleration $a_{w}^{G}$ in the global coordinate system. The mean value of each axis of $a_{w}^{G}$ was removed to reduce the impact of gravity.

### E. Movement Segmentation

#### 1) Anatomical Segmentation Method:

As discussed in Section I, the anatomical segmentation method decomposes continuous wrist movements into movement segments within the body’s anatomical axes. To achieve this, the gravity-free wrist acceleration $a_{w}^{G}$ was rotated to align with the subject’s body axes using the sternum sensor’s orientation $q_{L_{s}}^{G}$, as both share the same global coordinate systems. The resulting acceleration time-series, represented as $a_{w}^{L_{s}}$, was low-pass filtered to reduce high-frequency noise and then integrated in each axis using the trapezoid rule to yield the velocity times-series $v_{w}^{L_{s}}$. The integrated velocity $v_{w}^{L_{s}}$ was subsequently band-pass filtered using a 2^*nd*^ order Butterworth filter with cut-off frequencies of 0.1 *Hz* and 10 *Hz* to attenuate the low-frequency integration drift and high-frequency noise [37]. Movement segments were then obtained by decomposing $v_{w}^{L_{s}}$ at zero crossings along each axis independently. We removed movement segments with duration shorter than or equal to 50 *ms* or traveled distance less than 1 *mm* from further analysis [15], which are likely the result of sensor noise.

#### 2) Linear Segmentation Method:

The linear segmentation method leveraged two different sources of linear movements: actual linear movements annotated through video reviews and linear movements detected by a CNN. To train the CNN detection model, we segmented the local-coordinate wrist acceleration and angular velocity into 3-second sliding windows with 1/3 overlaps with adjacent windows [38]. A window was labeled as positive if it contained an entire linear movement based on the manual annotation. The CNN consisted of three convolutional layers, each with 30 filters of size 11. The output of each convolutional layer was passed through a ReLU activation and batch normalization, followed by a max pooling layer with a kernel size of 2 and stride of 2. The output of the second pooling layer was processed by a global average pooling layer and flattened. Finally, the flattened features were passed to a fully-connected layer, whose output was normalized using a Softmax function. The output of the CNN network was probability scores in the range of [0, 1], indicating the likelihood of containing linear movements for each window. To refine the predictions, we fine-tuned the decision boundary on the validation sets to maximize the precision score. The optimized decision boundary was then applied to the test sets to generate prediction scores for each test sample. Because a single linear movement could appear over multiple overlapping windows, temporally adjacent windows predicted to contain linear movements were merged into a larger, variable-length window. For robust evaluation, we employed the nested Leave-One-Subject-Out Cross Validation (LOSOCV) technique. LOSOCV is an evaluation method that is widely accepted as a means to generalize the performance of machine learning models with minimal biases, particularly for relatively small datasets [39]. The outer LOSOCV was used for model evaluation, while the inner LOSOCV within the training set focused on hyperparameter optimization.

The linear movement in each of the merged windows was then decomposed into movement segments. We first computed $v_{w}^{G}$ from $a_{w}^{G}$ by filtering and integration as described earlier. Then, we identified the dimension of the linear movement within 3D space by applying the Principal Component Analysis (PCA) to $v_{w}^{G}$, identifying the primary direction where the greatest variance of the movements is observed [17]. The movement segments were decomposed at zero crossings along this primary direction.

### F. Feature Extraction

We extracted kinematic data features from movement segments that were hypothesized to be relevant to motor deficits in stroke survivors [15]. We computed the mean, standard deviation, maximum, root mean square value, and coefficient of variation for the velocity in movement segments. Additionally, we calculated the mean absolute value and maximum value of the corresponding acceleration and jerk for each movement segment. Each movement segment’s absolute displacement and duration were also included. To capture the morphology of movement segments, we calculated the skewness, the number of peaks, speed metric (i.e., mean speed divided by peak speed), and jerk metric (i.e., negative mean absolute jerk divided by peak speed) [26]. The number of movement segments performed per second was computed as the total number of extracted movement segments divided by the duration. Finally, we aggregated these features of movement segments for each subject by calculating their mean, standard deviation, interquartile range, 10^*th*^, 50^*th*^, and 90^*th*^ percentiles. In total, 88 features were engineered. This feature extraction process was applied identically to the three movement segmentation methods.

### G. Unreliable Feature Elimination

To ensure a reliable estimation of motor deficits, we evaluated the test-retest reliability of the extracted features and removed those deemed unreliable. This step ensures that the subsequent machine learning algorithm leverages only reliable features, thereby generating a reliable output measure. Given that the data collection was cross-sectional, we divided the data into two groups to simulate a test-retest evaluation.

To assess feature reliability, we divided each subject’s entire time-series into halves temporally. For the anatomical segmentation method, features were independently computed from movement segments within each half. Similarly, for the linear segmentation method, linear movements from each half were processed to extract movement segments, from which data features were identically extracted. Feature reliability was quantified using the Intra-Class Correlation Coefficient (ICC(3,1)) between the data features derived from the two halves. Only reliable features with an ICC(3,1) greater than or equal to 0.75 [40] were kept for the subsequent machine learning training.

### H. Assessment of Motor Deficits Using Machine Learning

We employed a Multi-Layer Perceptron (MLP) to integrate reliable features into a measure quantifying motor deficits. Our algorithm was designed to ensure that the measure is both reliable and exhibits concurrent validity with weakly-labeled standards (i.e., clinician-evaluated assessments). Reliability was achieved by incorporating only reliable features into the machine learning pipeline, while the MLP was specifically tuned to address concurrent validity. To achieve this, we defined the loss function as $1-\overset{‾}{\rho }$, where $\overset{‾}{\rho }$ is the average Spearman correlation [41] with established upper-limb motor assessments (i.e., FMA-UE, negative median WMFT-PT, and WMFT-FAS). We opted to use only clinician-performed assessments to train the algorithm, excluding patient self-reported measures such as MAL-AoU and MAL-QoM, to maximize the objectivity of the assessment. The MLP architecture included an input layer and an output layer with a Sigmoid function, which normalized the output measure to the range of [0, 1]. The output measure estimated the motor status of stroke survivors with higher values representing better motor status.

We again employed nested LOSOCV for robust, generalizable evaluation. The learning rate for the Adam optimizer was determined via a logarithmically-spaced grid search in the range of [10*e*^−4^, 10*e*^−2^] within the inner LOSOCV. The batch size was 8, and the number of training epochs was 30. After identifying the optimal learning rate, the final MLP was trained on the entire training data designated by the outer LOSOCV. Subsequently, the trained model was evaluated on the left-out testing data. Model performance was evaluated by the Spearman correlation between the estimations and both clinician-performed and self-reported clinical measures.

To further verify the reliability of motor deficit assessment, the trained regression models were independently applied to two temporally isolated time-series of the testing subject. The resulting measures were then compared using ICC(3,1).

## III. Results

### A. Analysis on Motor Deficit Assessment

The anatomical segmentation methods extracted a total of 174,716 movement segments across the three axes of wrist-worn inertial data. In contrast, the linear segmentation method, using manually annotated linear movements, analyzed 14,946 movement segments—equivalent to only 8.6% of the total segments processed by the anatomical segmentation methods. The CNN-based linear movement detection method achieved a precision of 0.75 and a recall of 0.74 relative to manual annotations, identifying a total of 16,836 movement segments (9.6% compared to the anatomical segmentation).

All three segmentation methods extracted 88 features from their respective movement segments. The anatomical segmentation approach yielded 37 reliable features (42.05%) that were used to train the MLP model for motor assessment. In contrast, both linear movement segmentation approaches produced significantly fewer reliable features: 8 reliable features (9.09%) from actual linear movements and 5 features (5.68%) from automatically detected linear movements.

**Fig. 2.**
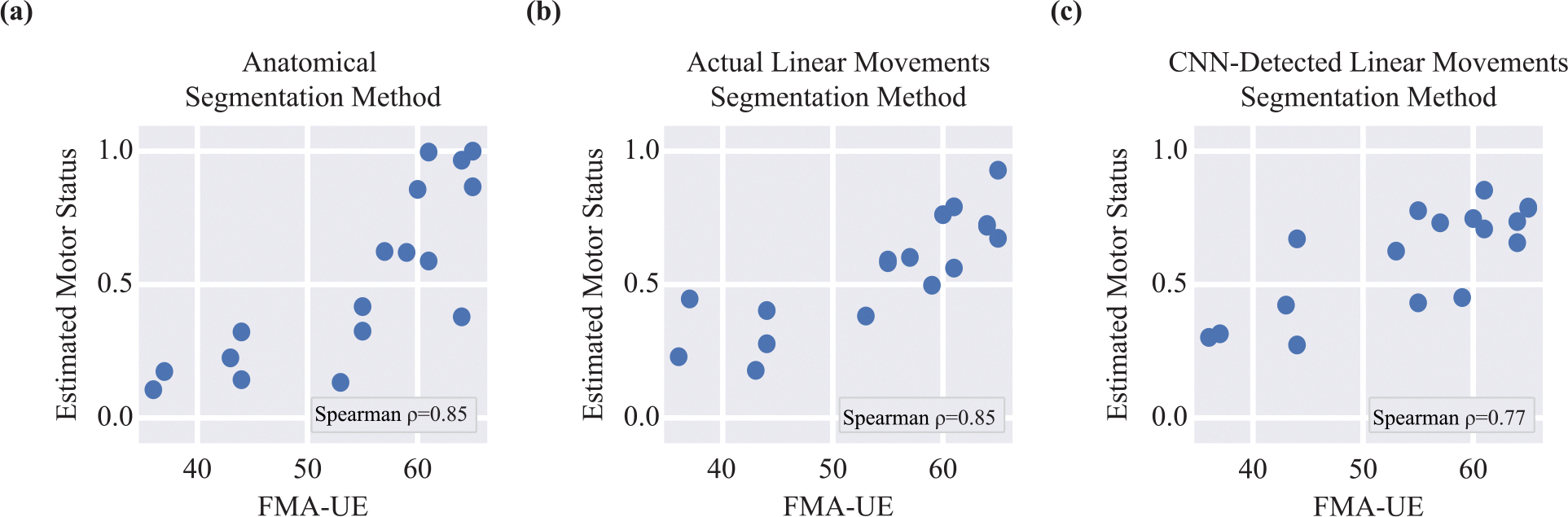
Associations between the FMA-UE, the most widely accepted assessment of motor impairment, and the proposed wearable-based motor assessment from (a) anatomical segmentation method, (b) actual linear movements, and (c) CNN-detected linear movements.

Table II summarizes the reliability and concurrent validity of the motor assessment for each segmentation method. All three methods showed acceptable reliability and concurrent validity against the primary outcome measure (FMA-UE), as ICC > 0.70 and correlation coefficient > 0.70 are deemed acceptable in medical measurements [42]. However, as illustrated in Fig. 2, the anatomical segmentation method and the linear segmentation method based on manual annotation demonstrated notably higher concurrent validity against FMA-UE compared to the linear segmentation method using detected linear movements. This difference is likely due to confounding errors from the misclassification of linear movements. A similar pattern emerged for validity against other clinical measures, as reflected by the average correlation coefficient.

Interestingly, Steiger’s z-test [43] did not reveal statistically significant differences in pairwise comparisons of Spearman correlation coefficients among the three segmentation methods. This lack of statistical significance may be a result of wide confidence intervals, as shown in Table II, likely caused by the relatively small sample size. Nevertheless, the overall pattern indicates that the linear segmentation method based on automatic detection performs less favorably than the other two methods.

### B. Analysis on Anatomical Segmentation Method

The anatomical segmentation method and the linear segmentation method based on manual annotation both analyzed wrist inertial data but relied on different coordinate systems rooted in distinct motor behavior theories, as detailed in Section II-E. However, two other key differences between these methods included the number of movement segments analyzed and the types of movements from which these segments were derived. The anatomical segmentation method incorporated 174,716 segments, covering both linear and non-linear movements, while the linear segmentation method includes only 14,946 segments, exclusively from linear movements from the primary direction. Consequently, we aimed to determine whether the quantity of data or the type of movement more significantly influences motor deficit assessment in anatomical segmentation, or whether performance is predominantly driven by the underlying theoretical frameworks.

First, to assess the effect of data quantity, we randomly sampled *p*% ∈ [10%, 100%] of movement segments identified by the anatomical segmentation method for each subject to develop the digital motor assessment. Notably, *p*% of movement segments were independently sampled from the first and second halves of the time series to estimate the reliability of the assessment. This process was repeated 5 times for each *p*% to minimize the effect of randomness. Second, to examine the effect of data type on motor deficit assessment, we divided the movement segments identified by the anatomical method into two groups: those derived from linear versus non-linear movements, as determined by manual annotations. Subsequently, the movement segments in each group were processed independently using the same data analysis pipeline to create a digital motor assessment.

**Fig. 3.**
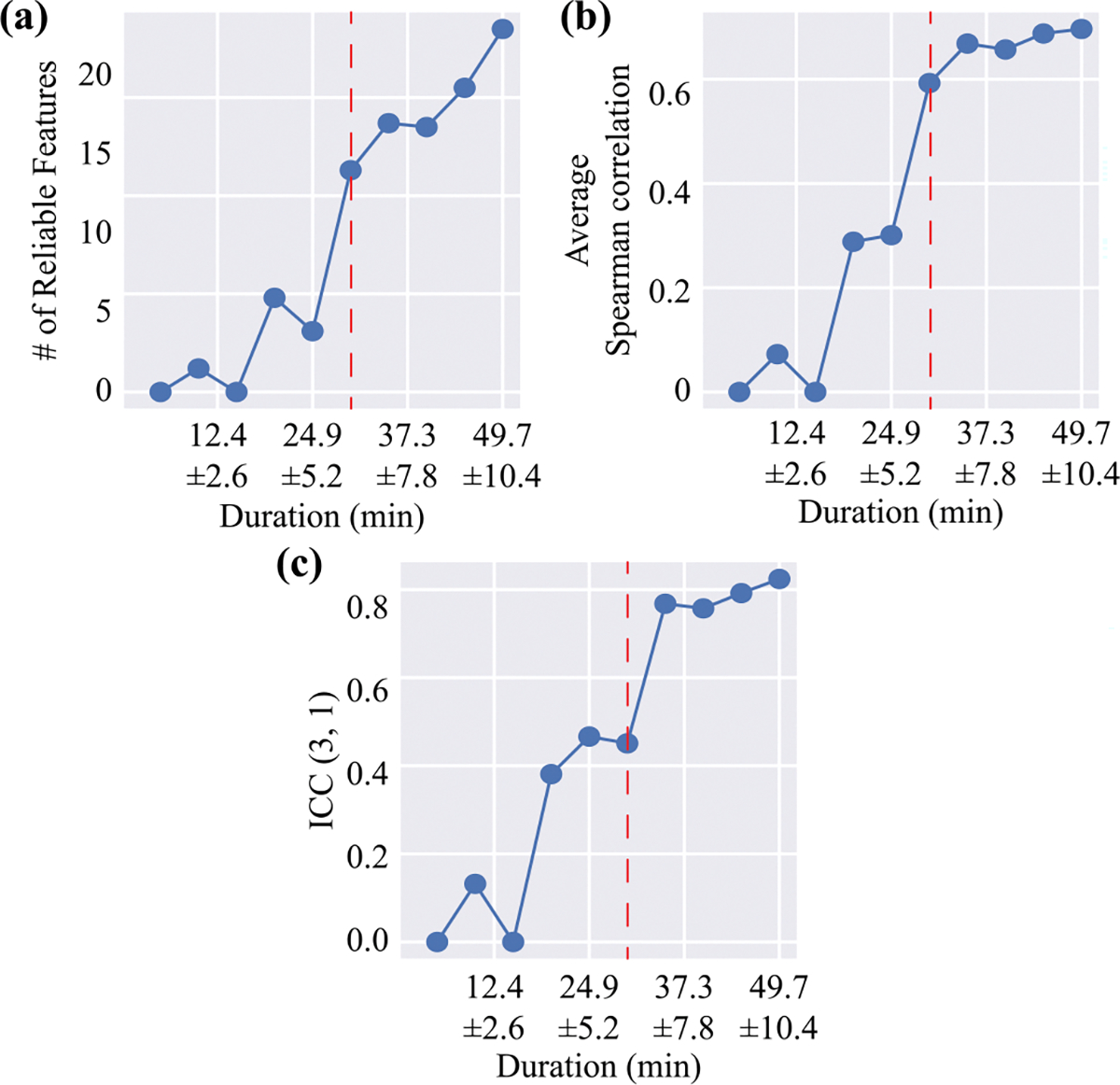
Performance of motor deficit assessment with varying numbers of movement segments extracted from the anatomical segmentation method. The figures illustrate the relationship between the percentage of movement segments included in training and (a) the number of reliable features extracted, (b) the average correlation between the estimated variable and all clinical measures, and (c) the reliability of the estimated variable. The red dashed line indicates 29.8 ± 6.2.

Fig. 3 summarizes how the number of movement segments impacts the extraction of reliable features, as well as the concurrent validity and reliability of motor assessments, for the anatomical segmentation approach. When fewer than 29.8 ± 6.2 minutes of data (approximately 104,829 out of 174,716 movement segments; 60%) were included, the number of reliable features for model training remained relatively small, as shown in Fig. 3a. Consequently, both the concurrent validity and reliability of the motor assessment were limited when fewer than 29.8 ± 6.2 of data were utilized. As the number of movement segments used to construct the digital motor assessment increased, so did the number of reliable features. However, concurrent validity and reliability plateaued at around 34.8 ± 7.3 minutes of data (approximately 122,301 out of 174,716 movements segments; 70%), as shown in Fig. 3b and c, indicating this threshold provided sufficient information for valid assessment. In contrast, the linear segmentation method based on manual annotation achieved strong reliability (ICC = 0.85) and concurrent validity (average correlation of 0.68), despite using substantially fewer movement segments in its analysis: 14,946 segments, corresponding to approximately 2.9 ± 1.7 minutes of linear movements.

Table III summarizes the effects of movement type (i.e., linear vs. non-linear movements) on the concurrent validity and reliability of anatomical segmentation. Among 174,716 movements segments, 54,652 were derived from linear movements and 120,064 from non-linear movements, as defined by manual annotations. The anatomical method, when applied solely to linear movements, yielded high reliability (ICC = 0.84) but relatively low concurrent validity (average ρ=0.60). In contrast, the linear segmentation method, which analyzed the same movement segments within the wrist’s local coordinate system, demonstrated comparable reliability but substantially greater concurrent validity (average ρ=0.68), as shown in Table II. This suggests that the theoretical frameworks underpinning these two segmentation methods are valid, particularly the idea that key information in linear movements is concentrated along the primary direction rather than general anatomical axes.

Conversely, applying the anatomical method to non-linear movements resulted in lower reliability (ICC = 0.70), likely due to the greater variability in how these movements are performed. However, it showed stronger concurrent validity (average ρ=0.71), possibly due to the greater number of movement segments included in the analysis, as observed in Fig. 3b. Combining both linear and nonlinear movements in the analysis leveraged the strengths of each type, yielding reliability close to that of linear movements (ICC = 0.82) while maintaining an average correlation similar to that of nonlinear movements (ρ=0.70).

### C. Analysis on Linear Movements Detected by CNN

Table II indicates that misclassification of linear movements in the linear segmentation method affects both the concurrent validity and reliability of the resulting outcome measure. Consequently, we seek to explore factors influencing linear movement detection performance, providing insights into how detection accuracy can be further enhanced.

**Fig. 4.**
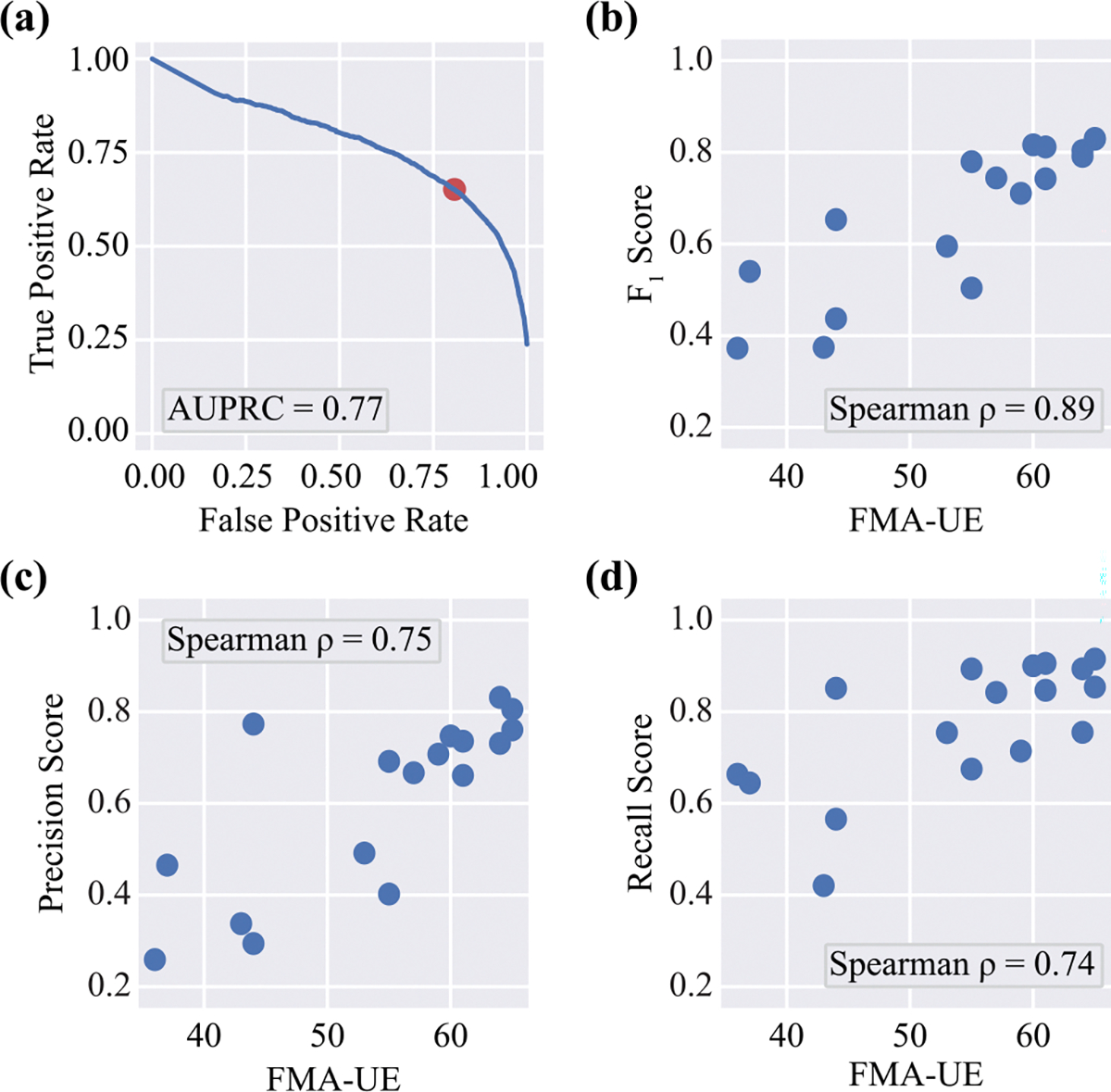
Performance of the CNN used to detect linear movements. (a) The Precision-Recall Curve. (b)-(d) The relationship between persubject *F*_1_, precision score, and recall score and FMA-UE, respectively.

The linear movement detection algorithm achieved an area under the precision-recall curve (AUC-PR) of 0.77 and an *F*_1_ score of 0.72 across study participants, as shown in Fig. 4a. At the individual level, the *F*_1_ score strongly correlated with FMA-UE scores, exhibiting a Spearman correlation coefficient of 0.89 (*p* < 0.01), as illustrated in Fig. 4b. To investigate whether detection performance was driven by precision or recall, we examined their relationships with FMA-UE scores. The results indicated strong correlations, with Spearman correlation coefficients of 0.75 for precision and 0.74 for recall, as shown in Fig. 4c and d, respectively. This suggests that detection performance decreases as motor impairment severity worsens. These findings align with prior research showing that more severe motor impairment leads to performance patterns that deviate further from normative patterns, complicating inertial pattern recognition [44]. Consequently, expanding data collection to include a more diverse cohort, particularly with moderately impaired individuals, could help improve linear movement detection performance.

We also investigated the influence of various CNN hyperparameters—including sliding window length, the number of convolutional layers, number of filters per layer, and kernel size—on detection accuracy measured by the *F*_1_ score. Results indicate that sliding window length and kernel size are the most critical parameters affecting performance, as detailed in Supplementary Materials Fig. S1. Additionally, we assessed the impact of training data size by varying the number of subjects included in the training set. As shown in Supplementary Materials Fig. S2, detection accuracy improved with larger training datasets, underscoring the need for expanded data collection.

## IV. Discussion

To the best of our knowledge, this is the first study to systematically examine the trade-off between the two emerging data processing approaches (i.e., anatomical vs. linear segmentation) for upper-limb motor assessment. We performed a comprehensive analysis to evaluate the effectiveness of each segmentation approach. Our findings demonstrated that the digital motor assessment derived from anatomical segmentation showed higher concurrent validity and reliability compared to the practical version of the linear segmentation approach, which relies on detected linear movements. However, the anatomical segmentation method requires an additional IMU sensor on the sternum, which can be cumbersome and reduce patients’ compliance in using the system continuously [33], [45].

In contrast, the linear segmentation method has the advantage of requiring only a single wrist-worn device, though at the cost of slightly lower concurrent validity and reliability. One factor that may influence this method’s effectiveness is the amount of linear movement data used in the analysis. In our study, linear movements performed by stroke-affected side accounted for only 2.9 ± 1.7 minutes out of 49.7 ± 10.4 minutes of collected data. We posit that the strong performance achieved, despite the limited data, is likely due to the information-dense nature of linear movements, as they are fundamental components of complex upper limb motions [25] and contain critical kinematic information related to motor severity [46], [47]. Nevertheless, extending data collection in real-world settings, such as monitoring throughout the day, could improve the validity of the assessment, which remains an important future study.

Another factor affecting the validity of the linear segmentation method is the accuracy of the linear detection model, as shown in Table II. However, developing a more robust model, such as a deeper CNN, would necessitate a substantially larger volume of labeled data, requiring extensive manual effort for video annotation. For instance, annotating one minute of video took an average of 30 minutes for a trained annotator in our study, posing scalability challenges. Consequently, there is a pressing need for novel approaches to automate the annotation process by analyzing video data [48] or synthesizing/augmenting human movement data [49].

It is worth noting that patients with similar clinical scores may exhibit varying digital assessment scores. For example, as shown in Fig. 2, less-impaired subjects (i.e., FMA-UE scores greater than 60) demonstrate considerable variability in their estimated motor status. This discrepancy comes from the fact that clinical scores primarily assess motor capacity (i.e., what patients can do) in controlled clinical settings [50], whereas our data collection was conducted in an environment that simulated real-world conditions, capturing patients’ actual motor performance (i.e., what they do in daily life). These two measures do not always directly translate into one another [51].

Our findings align with prior studies that employed anatomical and linear segmentation methods for assessing motor deficits in stroke survivors and patients with other neurological conditions. Wang et al. employed the linear segmentation method to create a digital assessment derived from longitudinal data collected outside clinical settings, achieving a Spearman coefficient of 0.86 between the digital outcome and the Action Research Arm Test (ARAT) [11], which is comparable to the results of our study (see Table II). Oubre et al. utilized the anatomical segmentation method using inertial data obtained during patients’ performance of pre-defined motor tasks. They reported a normalized root mean square error (NRMSE) ranging from 17% to 18.2% between the digital assessment and FMA-UE [15], [52]. In comparison, the NRMSE of our wearable-based assessment using the anatomical segmentation method with respect to FMA-UE was 21.7%. While comparable, we posit that the slightly higher error in our results stems from differences in study design: Oubre et al. used a predefined set of motor tasks, resulting in more homogeneous movements to analyze, whereas our study involved participants performing a diverse range of ADLs continuously, reflecting more complex and varied real-world scenarios. Datta et al. explored a variant of the anatomical segmentation method [16]. However, a direct comparison with our study is not feasible, as their work utilized the National Institute of Health Stroke Scale (NIHSS) to classify patients dichotomously into two categories: severe and mild-to-moderate motor severity.

We envision that the proposed wearable-based assessment, particularly using the linear segmentation method, could initially be used in inpatient settings, allowing patients to become familiar with the system. It could then be extended to outpatient settings for continuous monitoring of the motor recovery process, complementing standard therapy sessions. By continuously tracking patients’ responses to prescribed interventions, clinicians could refine treatment strategies to support more personalized rehabilitation [9]. Notably, personalized therapy that optimizes motor performance in real-world settings—rather than solely assessing motor ability in clinical environments—holds the potential for enhancing patients’ independence in daily life, the ultimate goal of rehabilitation [12]. Additionally, providing patients with access to their recovery data could empower their engagement in rehabilitation, facilitate collaborative communication with clinicians, and promote self-management of their condition [53].

This study has several limitations that warrant consideration. First, the sample size in this study was relatively small. To address this limitation, we employed nested LOSOCV for model training, validation, and testing. This method is widely recognized for its rigor and suitability in ensuring the robustness and generalizability of data-driven analyses with limited samples [39]. Nonetheless, future studies should include larger cohorts to further strengthen generalizability and enable the use of more advanced algorithms that may improve the accuracy of linear movement detection [54], [55], [56]. Second, the segmentation methods analyzed in this paper focus solely on wrist movements, overlooking hand dexterity, which is a crucial factor in differentiating impairment levels. While emerging evidence suggests that wrist-worn inertial sensors can capture some information about finger and hand movements through vibratory patterns [57], incorporating dedicated sensors, such as finger-worn accelerometers [33], [58], could further improve the accuracy of motor deficit assessment. Additionally, this study did not assess the responsiveness of each segmentation method to changes in upper-limb motor severity over time. Future work should incorporate longitudinal data collection to assess the responsiveness of these segmentation methods. Although previous studies indicate that stroke survivors tend to prefer wearing a single wearable devices on the wrist [33], [45], a limitation of our study is the lack of formal user or clinician feedback to directly assess comfort and practicality of the two segmentation methods. Future work will incorporate qualitative evaluations to better understand user experience and system usability across different sensor configurations.

## V. Conclusion

This paper is the first work that thoroughly examined anatomical and linear segmentation methods from a translational research perspective. It highlighted the trade-off between the complexity of data analysis and the usability of these two methods. Anatomical segmentation provides stronger concurrent validity against existing clinical measures but requires multiple sensors, which may cause inconvenience for continuous use. On the other hand, linear segmentation offers a more practical alternative as it requires only a single wrist-worn sensor and yields comparable estimations. However, its effectiveness in practice heavily depends on the accuracy of linear movement detection.

## Supplementary Material

supplemental

## Figures and Tables

**TABLE I:** Motor Tasks Resembling ADLs Performed by Study Participants

#	Motor Task	#	Motor Task

1	Unloading a grocery bag	7	Cleaning the table
2	Placing a tablecloth on a table	8	Folding bath towels
3	Preparing a sandwich	9	Brushing teeth
4	Drinking water from a cup	10	Writing on paper
5	Transferring the plate from a washing machine	11	Donning/doffing a coat
6	Mopping the floor		

**TABLE II:** Concurrent Validity and Reliability of Estimated Motor Status For Each Segmentation Method

Number of Inertial Sensors	Method	Concurrent Validity						Reliability
		Clinician-Performed Measures			Self-Reported Measures		Average Validity	ICC(3,1)
		FMA-UE	WMFT-PT	WMFT-FAS	MAL-AoU	MAL-QoM		
2	Anatomical Segmentation Method	0.86 (0.65, 0.95)	−0.57 (−0.83, −0.13)	0.78 (0.48, 0.92)	0.62 (0.19, 0.85)	0.65 (0.25, 0.86)	0.70	0.82 (0.58, 0.93)
1	Actual Linear Movements	0.85 (0.63, 0.95)	−0.54 (−0.81, −0.09)	0.82 (0.56, 0.93)	0.56 (0.11, 0.82)	0.60 (0.17, 0.84)	0.68	0.85 (0.64, 0.94)
	CNN-Detected Linear Movements	0.77 (0.46, 0.91)	−0.39 (−0.74, 0.11)	0.82 (0.56, 0.93)	0.59 (0.15, 0.83)	0.63 (0.22, 0.85)	0.64	0.81 (0.54, 0.93)

The 95^*th*^ confidence intervals for concurrent validity and reliability are included in the parentheses.

**TABLE III:** Concurrent Validity and Reliability of Anatomical Segmentation on Linear and Non-Linear Movements

Movement Type	Concurrent Validity						Reliability
	Clinician-Performed Measures			Self-Reported Measures		Average Validity	ICC(3.1)
	FMA-UE	WMFT-PT	WMFT-FAS	MAL-AoU	MAL-QoM		
Linear Movements	0.71 (0.36, 0.89)	−0.33 (−0.70, 0.18)	0.72 (0.36, 0.89)	0.59 (0.15, 0.83)	0.63 (0.22, 0.85)	0.60	0.84 (0.62, 0.94)
Non-Linear Movements	0.80 (0.52, 0.93)	−0.65 (−0.86, −0.25)	0.75 (0.43, 0.91)	0.65 (0.25, 0.86)	0.72 (0.36, 0.89)	0.71	0.70 (0.35, 0.88)

The 95^*th*^ confidence intervals for concurrent validity and reliability are included in the parentheses.

## Footnotes

1 Different terms have been used in prior studies to represent the identical concept, such as movement elements [15], [20] and submovements [18], [21].
