# Supplementary material for: Advancing Wearable-Based Upper-Limb Stroke Recovery Assessment to the Clinic: A Comparison of Movement Segmentation Strategies
Source: IEEE Trans Neural Syst Rehabil Eng. Author manuscript; Available in PMC 2026 Mar 4. (PMC12958275; doi:10.1109/TNSRE.2025.3635677)
Supplement: supplemental [file NIHMS2132336-supplement-supplemental.pdf]

Supplementary Materials

Table S1: The clinical scores of study participants in the study

Study Participant	FMA-UE	WMFT-PT	WMFT-FAS	MAL-AoU	MAL-QoM	Dominant Side	Impaired Side
1	43	2.88	3.67	2.29	2.57	Right	Left
2	55	1.57	4.40	2.00	2.79	Right	Left
3	57	4.19	4.07	1.15	1.30	Left	Left
4	36	4.68	3.00	1.78	2.09	Right	Left
5	44	2.88	3.67	1.53	1.33	Right	Right
6	55	1.88	4.60	3.80	3.60	Right	Left
7	64	1.47	4.93	3.24	2.72	Right	Left
8	53	3.69	4.33	3.36	2.97	Right	Right
9	65	1.59	5.00	5.00	4.52	Right	Right
10	37	3.81	2.87	2.50	2.08	Right	Left
11	61	1.87	4.73	4.02	4.03	Right	Right
12	44	6.50	3.67	3.13	2.33	Right	Left
13	59	1.40	4.53	4.92	4.35	Right	Right
14	64	2.03	4.67	4.83	3.82	Right	Right
15	61	1.85	4.73	3.82	3.21	Right	Right
16	65	1.84	5.00	4.43	3.91	Left	Right
17	60	1.75	4.67	4.50	4.56	Right	Left

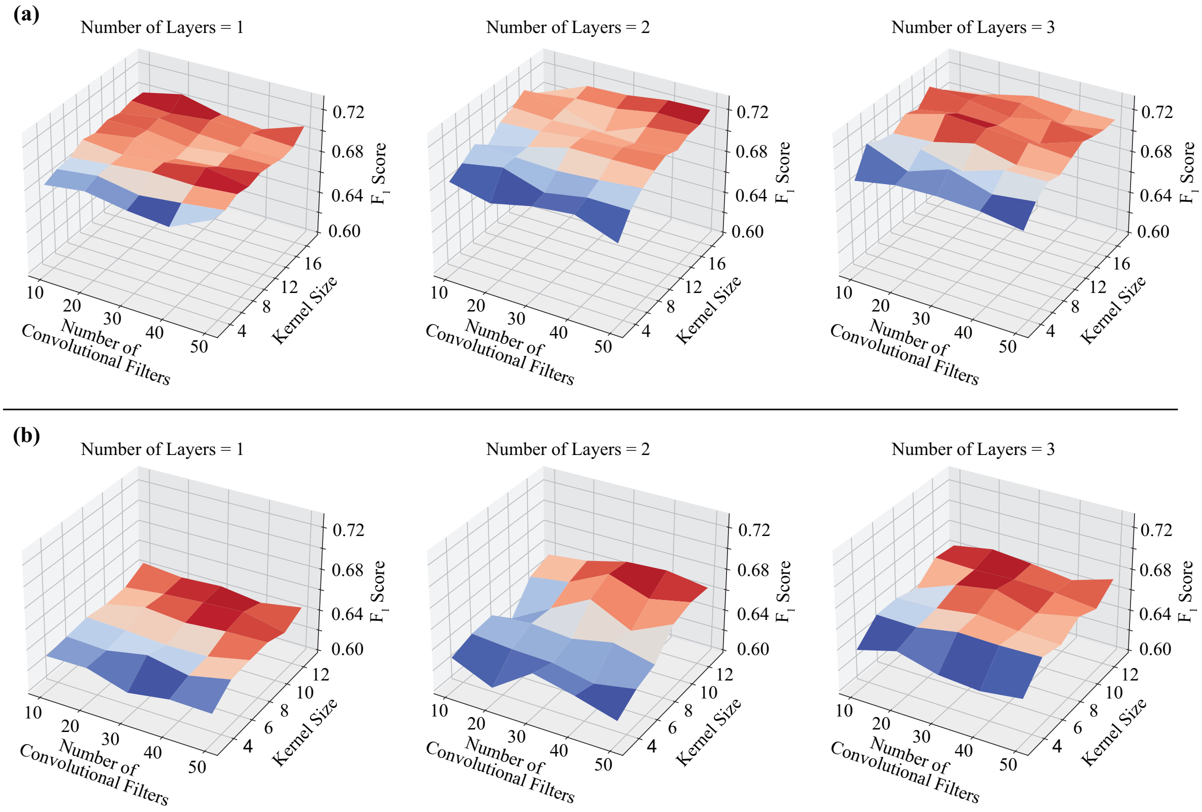

Figure S1: Ablation study results illustrating detection performance. (a) Detection accuracy measured by the F_1 score for models with one, two, and three convolutional layers using a 3-second sliding window. (b) F_1 scores for the same models but with a 2-second sliding window.

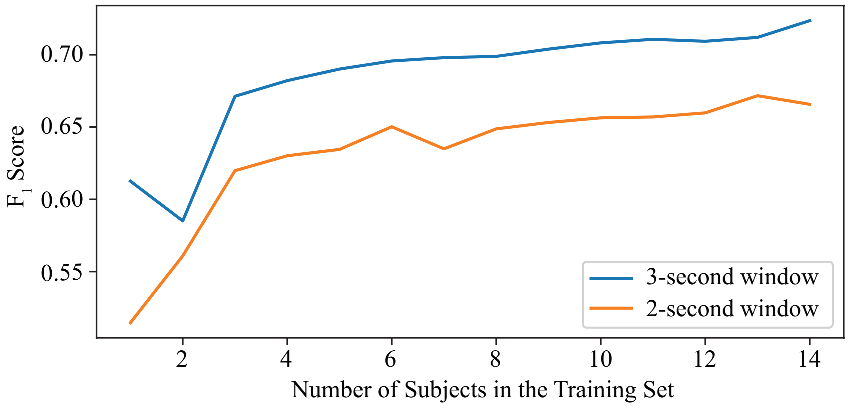

Figure S2: Linear movement detection model performance, measured by the F_1 score, plotted against the number of subjects included in the training set.
